# Respiratory energy demands and scope for demand expansion and destruction

**DOI:** 10.1093/plphys/kiac493

**Published:** 2022-10-22

**Authors:** Ulschan Bathe, Bryan J Leong, Kristen Van Gelder, Guillaume G Barbier, Christopher S Henry, Jeffrey S Amthor, Andrew D Hanson

**Affiliations:** Horticultural Sciences Department, University of Florida, Gainesville, Florida 32611, USA; Horticultural Sciences Department, University of Florida, Gainesville, Florida 32611, USA; Horticultural Sciences Department, University of Florida, Gainesville, Florida 32611, USA; Ginkgo Bioworks, Boston, Massachusetts 02210, USA; Mathematics and Computer Science Division, Argonne National Laboratory, Argonne, Illinois 60439, USA; Northern Arizona University Center for Ecosystem Science and Society, Flagstaff, Arizona 86011, USA; Horticultural Sciences Department, University of Florida, Gainesville, Florida 32611, USA

## Abstract

Nonphotosynthetic plant metabolic processes are powered by respiratory energy, a limited resource that metabolic engineers—like plants themselves—must manage prudently.

## Introduction

Photosynthesis is the primary energy input to plants, but most plant metabolic processes are powered much or all of the time by ATP and NAD(P)H that come from respiratory oxidation of photosynthetically produced carbohydrates, that is, “dark respiration” (Amthor, 1994). The processes of growth, nutrient uptake and assimilation, active transport, and maintenance are thus all clients of respiration and compete for their share of a respiratory energy budget that is capped by photosynthate income. The economic principle of opportunity cost applies to these clients’ competing demands: spending respiratory energy on process A means missing the benefit of investing that energy in process B (Mahmoudabadi et al., 2019). This principle is key to assessing prospects for crop improvement by metabolic engineering. As de Lorenzo (2015) put it: “metabolism…frames and ultimately resolves whether a given genetic program (existing…or engineered) can be deployed or not.” Foreign or reconfigured native processes bolted on to a crop-plant’s metabolic chassis must compete for respiratory energy with native ones without crashing the energy economy. A proviso on plant carbon budgets is that photosynthetic carbon fixation (“source activity”) can in certain cases increase to meet increased carbon demand (“sink activity”), that is, budget envelopes are not always fixed (Smith et al., 2018). However, as there is some consensus that productivity is most often limited or co-limited by carbon supply (Ainsworth and Long, 2005; Körner, 2015; Sonnewald and Fernie, 2018), we make this our basal assumption in the analyses below.

Despite its centrality, respiratory metabolism has always had far less attention in basic plant science and crop improvement than photosynthesis (Jacoby et al., 2016; Amthor et al., 2019)—a clear case of asymmetry in crop-focused research (Reynolds et al., 2021). This Update is a step toward symmetry. We first briefly recapitulate the classical basics of plant respiratory energy budgets and estimating the metabolic costs of various processes. We then apply these basics to assess how proposed synthetic biology/metabolic engineering interventions that add new demand or destroy unnecessary existing demand might affect yield. We also estimate the impact that these changes, if successful at scale, could have on the problems they set out to solve. Because sugar is the standard currency of plant metabolism, and convertible via fairly fixed exchange rates to the currencies of ATP and NAD(P)H, respiratory carbon budgets are usually reckoned in hexose equivalents for simplicity (Penning de Vries et al., 1974; Penning de Vries, 1975; Amthor, 1994; Loomis and Amthor, 1999). We follow this convention.

## Carbon budgets

**Figure 1:**
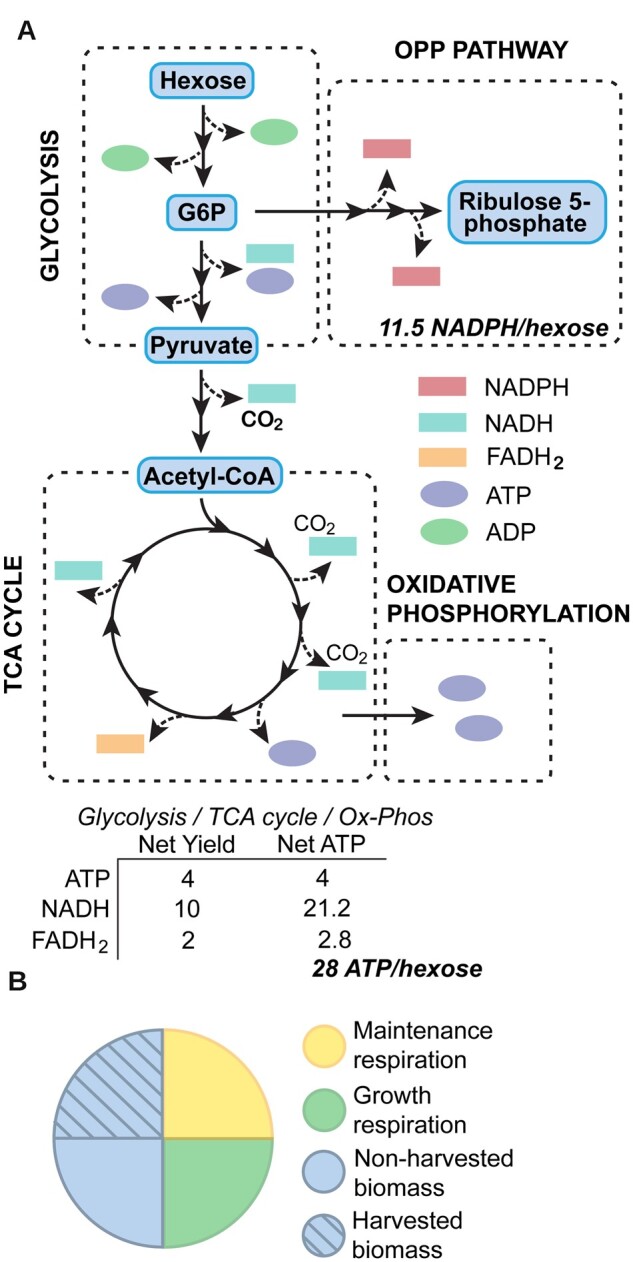
Plant respiratory metabolism and what it fuels. A, Overview of respiratory metabolism. B, The approximate fractions of total photosynthate that are respired to fuel growth or maintenance processes, or are allocated to harvested or nonharvested biomass. OPP pathway, oxidative pentose phosphate pathway; G6P, glucose 6-phosphate; Ox-Phos, oxidative phosphorylation; TCA cycle, tricarboxylic acid cycle.

The metabolic basis for budgeting in hexose units is that plant respiration can be abstracted as sequential oxidation of carbohydrates to CO_2_ via glycolysis, the pentose phosphate pathway, and the TCA cycle, with the TCA cycle coupled to the mitochondrial electron transport chain and oxidative phosphorylation of ADP (Figure 1A). Sucrose and starch are the main respiratory substrates; other substrates can feed into the TCA cycle but are ultimately derived from carbohydrates. Working in hexose units dovetails with analyses of photosynthesis based on sucrose or starch as end-product (Amthor, 2010). It does not account for direct use of “excess” ATP and reducing power from photosynthetic reactions (e.g. ATP use in chloroplast protein synthesis and electron flow to acceptors such as nitrite, sulfate, and oxaloacetate), but such uses are generally small, especially on a whole-plant basis (Eichelmann et al., 2011; Tcherkez and Limami, 2019). The rate of respiration may be somewhat lower in light than darkness in photosynthetic cells, but this effect is hard to measure, and again small on a whole-plant basis (Gauthier et al., 2020). Respiration is usually measured as CO_2_ release or O_2_ uptake (in the dark for photosynthetic cells); nonrespiratory reactions contribute little to this gas exchange.

Respiration delivers three main products needed for growth and survival: (1) pathway intermediates providing carbon skeletons for anabolic pathways; (2) ATP from substrate-level and oxidative phosphorylations; and (3) reducing power (NADH or NADPH) (O’Leary et al., 2019). The ATP yield of complete respiration of a hexose in plants is not fully settled (Jacoby et al., 2015); we use a conservative value of 28 ATP/hexose, corresponding to a P/O ratio of 2.33. Plant alternative oxidase often appears to be at least modestly active (Cheah et al., 2014), in which case 28 ATP/hexose would not be attained and hexose costs of a process increase, that is, our costs are minimum ones. NADPH is the main reductant for plant anabolism; respiration can theoretically produce ∼11.5 NADPH/hexose via the oxidative pentose phosphate pathway (Williams et al., 1987; Amthor, 2010); we use this “best-case” value and apply it to NADH also.

The carbon skeletons, ATP, and NAD(P)H generated by respiration together support the full range of growth and maintenance processes. In very broad terms, for an annual crop: (1) around half the carbon assimilated by photosynthesis (net of photorespiration) is subsequently respired as CO_2_ (range 30%–60%; Table A1 in Amthor, 2000; Amthor et al., 2019); and (2) this CO_2_ release is about evenly split between respiration that supports growth and respiration that supports maintenance (Figure 1B; Amthor, 1989; Amthor et al., 2019). This rough but robust estimate of the fractions of photosynthate consumed by growth and maintenance respiration provides context for our assessments of the opportunities to reduce these demands or to divert respiratory carbon and energy to beneficial new demands.

## Energy costs of biomass synthesis

**Figure 2:**
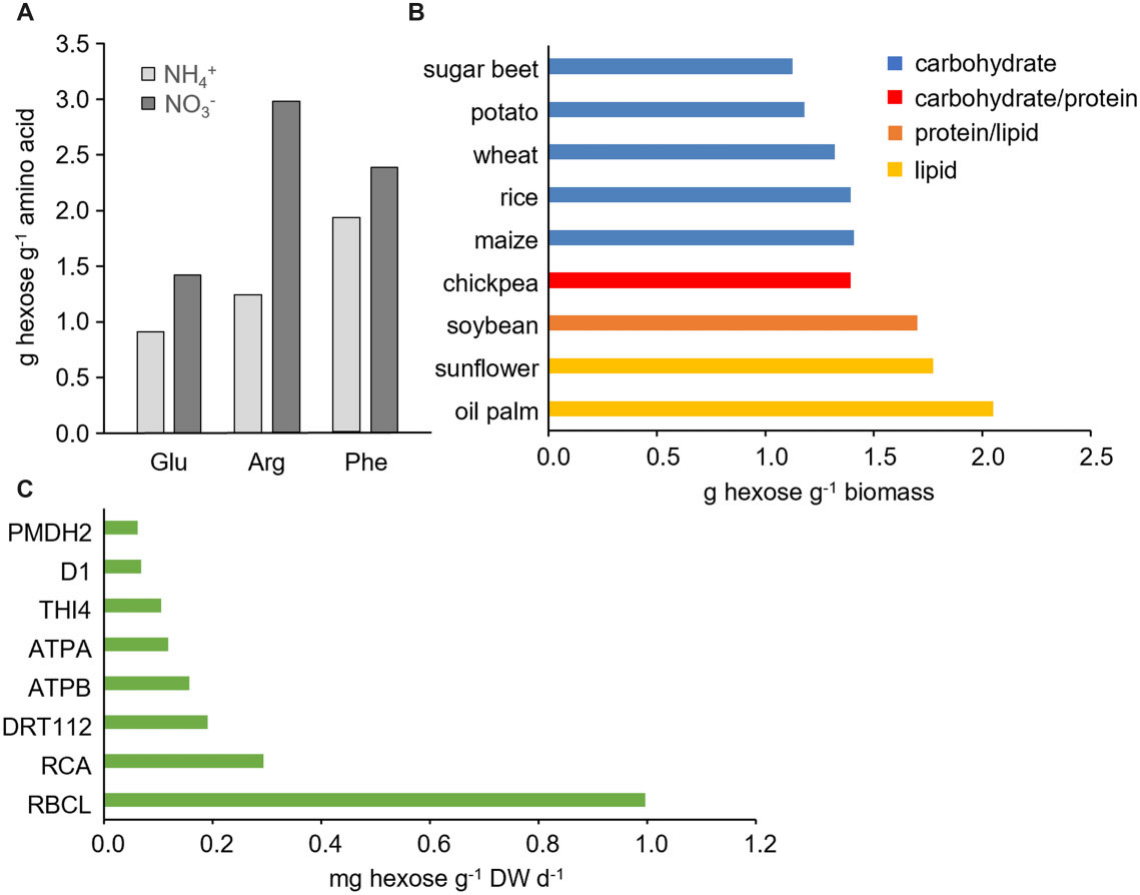
Energy demands of growth processes and protein turnover. A and B, grams of hexose required to make 1 g of product at maximum theoretical efficiency of biosynthesis. A, Hexose costs, with ammonium or nitrate as N source, for three progressively more expensive amino acids. Biosynthetic pathway analysis from Amthor (2010), using 28 ATP/hexose, 11.5 NAD(P)H/hexose, and nitrate uptake and reduction costs as in Supplemental Appendix S1. B, Hexose costs of various crop storage organs: sugar beet roots, potato tubers, inflorescences with seeds (rice, wheat, and sunflower), maize cobs (70% seeds), pods with seeds (chickpea and soybean), and oil palm fruits. Carbohydrate, protein, lipid, lignin, organic acid, and mineral concentrations in organs are based on Penning de Vries et al. (1983). Substrate requirements for biosynthesis of each class of constituent are from Penning de Vries et al. (1983) and Amthor (2010) (for lignin and carbohydrates). Tool maintenance estimates are included, and 5% of the carbohydrate substrate is respired to provide energy to transport the other 95% from sources to storage organs. Minerals are supplied as needed, accounting for uptake cost in roots. The N source is nitrate and its assimilation cost is included. C, Hexose costs of breakdown plus resynthesis of eight Arabidopsis leaf proteins, recalculated from data of Li et al. (2017). DW, dry weight.

Plant growth can be abstracted as the conversion of carbohydrate—plus nitrogen (N), sulfur, phosphorus, and other elements—to new biomass. The theoretical upper limit on efficiency of converting hexose to biomass components can be calculated from input-output analysis of biosynthetic pathways plus the hexose oxidized to provide any additional ATP or reductant required for biosynthesis, including that for polymerization, transport of substrates from source sites to growth sites, and construction and maintenance of biosynthetic machinery (Penning de Vries et al., 1974; Amthor, 2010; Arnold et al., 2015). The efficiency (mass-for-mass) of converting hexose to biomass depends strongly on the chemical makeup of the new biomass—especially on how chemically reduced its constituents are, and on the N source (nitrate or ammonium). Thus, with ammonium as N source, it takes more hexose to make arginine or phenylalanine than to make glutamate (Figure 2A) because arginine and phenylalanine both require more carbon atoms and ATP than glutamate. All three amino acids require more hexose when nitrate is the N source due to the ATP cost of taking nitrate up and the NAD(P)H cost of reducing nitrate to ammonium (Supplemental Appendix S1); the increased hexose demand is greatest for arginine because it has four N atoms while the others have only one (Figure 2A). Similarly, at an aggregate biomass level, more hexose is needed to make high-protein or high-lipid biomass compared with high-carbohydrate biomass due to the ATP and reductant costs of amino acid and fatty acid biosynthesis (Figure 2B). To make biomass construction costs easily accessible for research and teaching, we built a KBase app that calculates the hexose cost of biomass for a user-specified composition:https://narrative.kbase.us/#catalog/apps/QuantitativePlantAnalysis/compute_plant_biomass_yield.

Growth respiration rate is temperature-dependent only to the extent that growth rate itself is, because growth respiration is driven by near-constant, temperature-independent stoichiometries of the biosynthetic pathways multiplied by the growth rate. The scope to reduce growth respiration without changing biomass composition is limited as most plant biosynthetic pathways are already quite efficient (Amthor et al., 2019) and installing novel, more-efficient pathways is intrinsically difficult (Erb et al., 2017).

## Energy costs of biomass maintenance

Maintenance respiration for herbaceous plants is typically 10–30 mg hexose per gram plant dry mass per day at normal temperatures, as estimated by measuring respiratory CO_2_ release at various growth rates and taking the extrapolated rate at zero growth as the maintenance rate (Table 3 in Penning de Vries, 1975; Amthor, 2000; Amthor et al., 2019). Maintenance respiration is less metabolically understood than growth respiration, but its main drivers are clearly turnover (i.e. breakdown and resynthesis), primarily of enzymes, and active transport processes that counter various leaks and maintain ionic balances. Maintenance respiration changes in response to short-term (minutes to hours) temperature change with a Q_10_ of ∼2, and with protein content, proteins being subject to faster—and more costly—turnover than other major biomass constituents (Penning de Vries, 1975; Amthor, 2000). Protein turnover likely accounts on average for roughly half of maintenance respiration (see the lowball estimate of 43% ± 22%, mean ± standard deviation, Supplemental Table S1). The ATP (and hexose) cost of protein turnover can be estimated from measured turnover rates and calculated values for ATP requirements of degradation and resynthesis (see Figure 2C for examples of protein turnover costs). We use per-amino acid residue values of 1.3 ATP for degradation and 5.0 ATP for resynthesis (de Visser et al., 1992; Amthor, 2000); 5.0 ATP is the cost of repolymerization alone, that is, it is based on zero catabolism and resynthesis of the amino acids and thus represents the lower limit of resynthesis cost. We disregard organellar protein import costs, which are nonnegligible but are quantitatively ill-defined in vivo (Chen et al., 2019).

## Costing engineering interventions

The demand-expansion and demand-destruction interventions below involve energy and carbon fluxes large enough to substantially affect yield, that is, the interventions add or subtract sizeable energy and/or carbon sinks. In each case, we attempt to assess agronomic/economic potentials and tradeoffs using the basal assumptions that changing the sink does not alter photosynthetic source activity (see above) and that biomass composition does not change beyond our explicit alterations.

## Energy demand expansion

### Installing recalcitrant polymers for carbon sequestration

**Figure 3:**
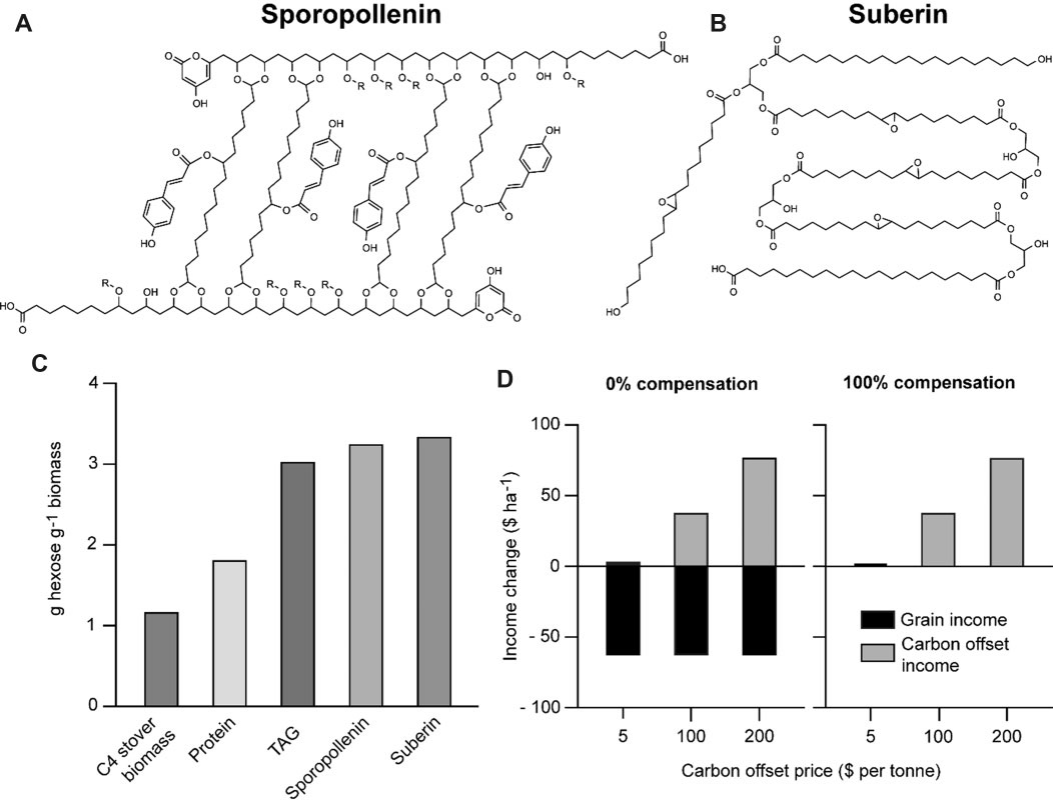
Potential root-synthesized, recalcitrant CCS polymers and their economics. A, The molecular structure of sporopollenin, based on pine sporopollenin analysis (Li et al., 2019). R is glycerol. B, The molecular structure of suberin (Pereira, 2015). C, Calculated hexose costs for synthesis of typical C4 stover biomass, protein, triacylglycerol (TAG, specifically triolein), sporopollenin, and suberin, based on Amthor’s values (2010) and Supplemental Table S2. D, Estimated impacts on farm income per hectare of engineering maize roots to make extra suberin or sporopollenin equal to 10% of root dry weight, in two scenarios (0% or 100% compensation of the added carbon and energy demand by increased photosynthesis or decreased respiration). Grain price is $166 per tonne; carbon offset price is $5, $100, or $200 per tonne of CO_2_.

Carbon capture and storage (CCS) using agricultural technologies that enhance soil carbon storage (“carbon farming”) is anticipated to contribute importantly to the net negative emissions goal of 10 Gt of CO_2_ equivalents per year by 2050 (National Academies of Sciences, Engineering, and Medicine, 2019; Bomgardner and Erickson, 2021). One such technology is to channel a portion of a crop’s photosynthate to deposition in roots as biodegradation-resistant (“recalcitrant”) compounds that are long-lived in soil. Annual root production then drives up the stable organic carbon content of soil, effectively sequestering CO_2_ from the atmosphere on a long-term basis (Busch and Miller, 2022), for which the farmer is paid a “carbon offset” price. The current global average carbon offset price is ∼$4 per tonne of CO_2_ (∼$15 per tonne of carbon) but prices vary greatly between nations and regions (e.g. ∼$37 per tonne currently in Canada) and are projected to rise in the next decade (World Bank, 2022). The preferred compounds are polymeric, highly reduced, and contain no N or sulfur (which are energetically expensive to assimilate and, if in limited supply, would constrain the compounds’ production). Two compounds that have become targets for engineering long-lived soil carbon sinks are suberin (Busch and Miller, 2022) and sporopollenin (Glinkerman et al., 2021) (Figure 3, A and B). Suberin occurs naturally in walls of root and bark cells (Woolfson et al., 2022), and sporopollenin in walls of pollen grains and spores (Grienenberger and Quilichini, 2021). Both compounds are resistant to degradation in soil (Mackenzie et al., 2015; Angst et al., 2016). Their half-lives in soil are presumptively on the decades-to-centuries timescale needed for effective carbon sequestration (Powlson et al., 2011) although field data on this are lacking. Beyond sequestering atmospheric CO_2_, accumulation of soil organic carbon can benefit crop health and productivity (Powlson et al., 2011). However, as normal soil organic matter is mainly microbial necromass (Dynarski et al., 2020), it is not a priori obvious that undegraded plant polymers would bring the same benefits, and field data are lacking on this point also.

Average structures of sporopollenin and suberin (Figure 3, A and B) can be used to calculate their biosynthetic costs, which we do in the context of a maize (*Zea mays*) crop (Figure 3, C and D; Supplemental Table S2). The costs of both polymers, at ∼3.3 g hexose g^−1^, are similar to triacylglycerol (reflecting their mainly lipid nature), and far more expensive than maize grain (1.41 g hexose g^−1^, Figure 2B) or stover (1.19 g hexose g^−1^, Figure 3C). If adding a suberin or sporopollenin sink is not compensated by increased photosynthesis or decreased respiration (basal scenario), it follows that each gram of extra suberin or sporopollenin accumulation engineered in a maize plant’s roots would reduce the plant’s grain yield by (∼3.3/1.41) ≈2.4 g if grain filling is the only process from which photosynthate is diverted. A best-case scenario would be that the extra sink demand is matched by an increase in photosynthesis (Smith et al., 2018) or decrease in respiration (see section “Demand destruction”) so that the extra suberin or sporopollenin imposes no yield penalty. We now use these two scenarios (i.e. zero or 100% compensation by increased photosynthesis or decreased respiration for the extra suberin or sporopollenin sink) to assess the potential farm-level economics of CCS using maize engineered to accumulate a reasonable amount of suberin or sporopollenin in roots.

This assessment assumes a root dry mass of 1.6 tonnes ha^−1^ (Ma et al., 2003), an engineered addition of suberin or sporopollenin equal to 10% of root dry mass (i.e. 0.16 tonnes ha^−1^, equivalent to 0.53 tonnes ha^−1^ hexose consumed), a maize grain yield of 11.2 tonnes ha^−1^ (178 bushels acre^−1^), and a grain price of $166 per tonne (Schnitkey et al., 2022a, 2022b). The zero compensation case predicts a grain yield reduction of 0.38 tonnes ha^−1^, decreasing farm income by $64 ha^−1^. As suberin and sporopollenin are ∼66% carbon by weight, 0.16 tonnes of either corresponds to ∼0.11 tonnes of carbon, or ∼0.39 tonnes of CO_2_. At current carbon offset prices, sequestering this much CO_2_ would generate $1.60 ha^−1^, that is, far too little to replace revenue lost from reduced grain yield. Offset prices would have to rise to ∼$200 per tonne of CO_2_ for this scenario to become profitable (Figure 3D). At current carbon prices, the 100% compensation case gives $1.60 ha^−1^ extra income (Figure 3D), but this is only 0.1% of that from grain sales. Again, far higher carbon offset prices would be needed for robust profitability.

Combining the above numbers with the recent average US maize crop area (34 million ha; USDA, 2022) indicates that the CCS potential of engineering the entire US maize crop to accumulate 0.16 tonnes ha^−1^ of suberin or sporopollenin in roots would be 13 million tons of CO_2_ per year. This is 0.3% of the annual US fossil CO_2_ production (∼4.1 billion tonnes; EPA, 2020).

These calculations illustrate three points about prospects for engineered suberin and sporopollenin as CCS technologies. First, these interventions could entail substantial yield penalties unless they are mitigated by increased photosynthetic carbon gain or decreased respiratory carbon loss. Second, the higher the carbon offset price, the more economically viable the technologies become. Third, they can contribute only modestly to overall atmospheric carbon drawdown.

### Replacing the Haber–Bosch process with BNF

**Figure 4:**
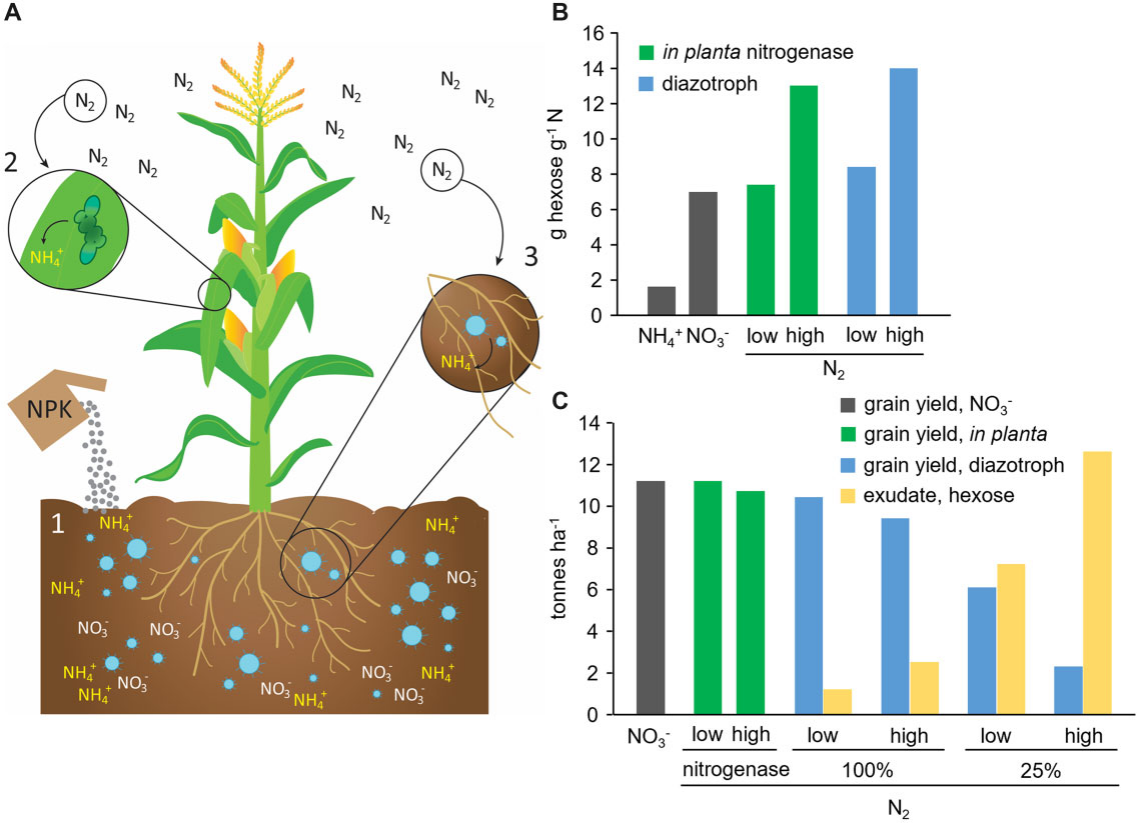
Obtaining N from in planta BNF or diazotroph BNF instead of fertilizer nitrate. A, Potential sources of N for maize: 1, Soil nitrate (or ammonium) from fertilizer, taken up by roots; 2, Nitrogenase system engineered in leaves; 3, Soil diazotrophs that transfer fixed N to the plant via the roots. B, The hexose cost of N (assimilated into glutamate) when the source is soil nitrate, soil ammonium, or atmospheric N_2_. N_2_ costs are calculated for an engineered leaf nitrogenase (in planta nitrogenase) and for BNF by root-associated diazotrophs (diazotroph), and for low or high ATP requirements (16 or 40 ATP per N_2_, respectively). C, Estimated maize grain yields for crops in which BNF replaces all the N (120 kg ha^−1^) that a benchmark crop obtains from nitrate, and the amounts of photosynthate (as hexose) exuded from roots that would be needed to support diazotroph BNF. Calculations assume diazotrophs use either 100% or 25% of the exuded hexose for N fixation.

In the century since its invention, the Haber–Bosch industrial process for synthesis of ammonia from H_2_ and N_2_ has grown to the point that it now fixes ∼120 million tonnes of N per year. This is double the fixation by natural terrestrial sources (Fowler et al., 2013) and feeds half the world population (Erisman et al., 2008). Besides its great benefits, Haber–Bosch N use has negative environmental consequences, especially water and air pollution (Robertson and Vitousek, 2009; Fowler et al., 2013), and these have led to much interest in supplementing—or even replacing—Haber–Bosch N with different versions of biological N fixation (BNF). These versions include transferring bacterial nitrogenase systems to crop genomes (Good, 2018) and harnessing native or engineered N-fixing bacteria (diazotrophs) living free in the root zone or as endophytes (Mus et al., 2016; Wen et al., 2021). We consider the energy and carbon costs of these forms of BNF using maize as a representative cereal crop and benchmarking the costs against nitrate as N source, with 120 kg ha^−1^ N from soil nitrate being entirely replaced by BNF (Figure 4A). The parameters and assumptions used are given in Supplemental Appendix S1.

The direct biochemical costs of acquiring N and assimilating it via ammonium into amino acids differ greatly among the three main forms of N potentially available to plants (Figure 4B; Supplemental Appendix S1). Ammonium is cheapest because it needs no reduction and does not necessarily require energy for uptake (Forde and Clarkson, 1999). Nitrate—the most important soil source of plant N—is several-fold more expensive because it needs energy for uptake and reduction (see Figure 2A). Explaining its enduring attraction despite its huge biochemical challenges (Vicente and Dean, 2017), BNF by a nitrogenase system engineered in leaves could theoretically cost only slightly more than nitrate if reduction of one mole of N_2_ is reckoned to take 16 moles of ATP, as it does in vitro (Seefeldt et al., 2018). The same applies to BNF by plant-associated diazotrophs, which has a small extra cost for transfer of the fixed N to the plant. However as the ATP requirement in vivo can rise to 40 moles per mole of N_2_ (Bueno-Batista and Dixon, 2019), BNF could also cost much more. We therefore explore two BNF scenarios for a maize crop, based on low (16) or high (40) ATP demands. These scenarios do not include the costs of synthesizing and maintaining a nitrogenase system in the leaf or the costs of maintaining the diazotroph population, both of which can be high.

For simplicity, we assume that diazotroph BNF is fueled by sugar (hexose) exuded by roots, that this hexose comes out of a fixed plant carbon budget at the expense of grain yield (set as above at 11.2 tonnes ha^−1^ when nitrate is the N source), and that it takes 1.41 g of hexose to produce 1 g of grain (see Supplemental Appendix S1; Figure 2B). For diazotroph BNF, we further assume either (1) that all the hexose exuded by roots is taken up by diazotrophs and that the nitrogenase system uses all of it (“100%” case), or (2) that only half is taken up and that the nitrogenase system uses only half of this half (“25%” case). The 100% case is highly optimistic and surely underestimates the hexose demand and the yield penalty. The 25% case is more realistic (Mus et al., 2016), but may still tend to optimism. In every case, we assume that all of the N fixed by the diazotroph is transferred to the crop; this assumption is again highly optimistic.

The grain yield penalties in these scenarios, relative to a maize crop with nitrate as N source, range from almost zero (in planta leaf nitrogenase system, low ATP demand) to 79% (diazotroph BNF, high ATP demand, 25% of exuded hexose used by nitrogenase system) (Figure 4C). The lowest-cost diazotroph BNF scenario predicts a yield loss of 0.5 tonne ha^−1^, which would seem to conflict with an average 0.35 tonne ha^−1^ yield gain in maize crops treated with a *Klebsiella variicola* strain engineered to enhance BNF (Wen et al., 2021). However, these numbers can be reconciled. Our cost estimates are for BNF to replace *all* the crop’s fertilizer-origin N (120 kg ha^−1^) whereas the *K. variicola*-treated crops also received normal fertilizer N applications, that is, BNF likely contributed at most a few tens of kg N ha^−1^ to the crop N budget. Further, *K. variicola* has growth-promoting activities besides BNF (Yang and Yang, 2020) that could have boosted yield. More generally, 120 kg N ha^−1^ is far more than diazotrophs typically provide to crops, for example, an estimate for sugar cane (*Saccharum officinarum*) is ∼25 kg ha^−1^ year^−1^ (Herridge et al., 2008).

Predicted amounts of sugar exudate needed to fuel diazotroph BNF in our scenarios range from 1.2 to 12.6 tonnes ha^−1^ (Figure 4C). To set these amounts in context: *total* carbon losses from roots (of which exuded sugars and other bioavailable solutes are merely a fraction) are typically ∼20% of net photosynthate (Hütsch et al., 2002; Nguyen, 2003), which in our model maize crop would be 24 tonnes ha^−1^, assuming a harvest index of 0.5 (Hütsch and Schubert, 2017) and 1.6 tonnes ha^−1^ of roots (Ma et al., 2003). Hence, *total* carbon loss from the maize roots is probably no more than ∼5 tonnes ha^−1^. Thus, based on our assumptions, BNF supported by root exudates seems unlikely to be able to replace more than a small fraction of the N currently supplied by fertilizers.

## Energy demand destruction

### Scope for slowing enzyme protein turnover

As said at the outset, ∼50% of crop respiratory energy goes to support maintenance processes (Figure 1B), and roughly half of that 50% fuels turnover of enzymes and other proteins (Supplemental Table S1). Besides being governed by developmental stage, environmental conditions, misfolding, and general nonenzymatic damage reactions such as carbonylation and nitrosylation (Nelson and Millar, 2015), enzyme turnover can be driven in a more specific way by self-inactivation resulting from chemical damage done to the enzyme by its own reaction mechanism, substrates, or products (Bathe et al., 2021; Hanson et al., 2021; Colinas and Fitzpatrick, 2022). Extreme examples are suicide enzymes, which self-inactivate after mediating a single catalytic cycle, for example, the thiazole synthase THI4 (Joshi et al., 2020). Less extreme cases are enzymes that have radical mechanisms (e.g. the phosphomethylpyrimidine synthase THIC) or chemically reactive substrates or products (e.g. histidinol dehydrogenase and tryptophan synthase) (Hanson et al., 2021). The short working lives of such enzymes become apparent when the metric Catalytic Cycles till Replacement (CCR) is applied to them (Tivendale et al., 2020; Hanson et al., 2021). CCR is the number of catalytic cycles that an enzyme mediates in vivo before being degraded, that is,


(1) $$\mathrm{CCR}=\frac{\mathrm{Metabolic}\;\mathrm{flux}\;\mathrm{rate}}{\mathrm{Enzyme}\;\mathrm{replacement}\;\mathrm{rate}}.$$

Estimating CCR values has recently become possible thanks to advances in proteomics and in flux analysis and modeling (Tivendale et al., 2020). A suicide enzyme like THI4 has a CCR of one and very short-lived enzymes have CCRs of tens to hundreds, but CCRs in Arabidopsis (*Arabidopsis thaliana*) range up to >10^7^ and the median value is 4 × 10^5^ (Hanson et al., 2021). The high energy cost of protein turnover (≥6.3 ATP per residue, see above) makes abundant enzymes with low CCRs particularly large items in the maintenance energy budget, and hence prime targets for energy demand destruction by extending their working lives (Bathe et al., 2021). There may well be considerable scope for such life-lengthening.

First, plant enzymes may never have been under strong natural selection to minimize protein turnover costs because, during evolution, plants generally had surplus carbohydrate that could not be used for growth due to lack of N and other nutrients (LeBauer and Treseder, 2008; Körner, 2015; Prescott et al., 2020). The same applies to selection pressure in crop breeding, at least prior to the 20th century when the Haber–Bosch process, industrial agriculture, and scientific breeding arrived. Crop ancestors and, throughout most of their evolution, crops themselves may thus have had excess carbohydrate to spend on needlessly fast protein turnover, meaning that turnover rates of today’s crop enzymes—and hence maintenance respiration rates—may be reducible. Certain reports of progressive reduction in crop respiration rates support this possibility (e.g. McCullough and Hunt, 1989; Earl and Tollenaar, 1998). Second, such improvement has been demonstrated; certain natural enzymes have residues favoring self-inactivation that can be mutated to other residues that extend life but hardly affect catalytic activity (Bathe et al., 2021). Third, the lifespan of low-CCR enzymes prone to catalytic self-inactivation can in principle be lengthened via continuous directed evolution in yeast or *Escherichia coli* since damage that an enzyme does to itself will occur whether the enzyme is in its native host or a foreign host (Hanson et al., 2021; García-García et al., 2022). The long-life mutations thus obtained could then be made in a target crop by base-editing or gene replacement (Gionfriddo et al., 2019). Testing if enzyme life-extension can be done in practice is thus a future priority (see “Outstanding Questions”).

### Potential crop yield gains from slowing protein turnover

**Figure 5:**
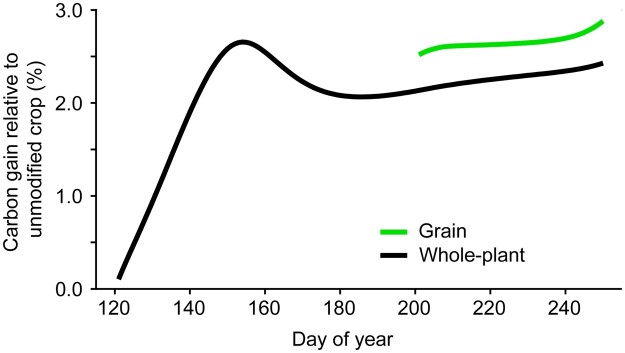
Predicted impact of reduced protein turnover cost on crop yield. Percentage gain in whole crop biomass carbon accumulation and grain carbon storage for a crop with 6.5% reduction in maintenance respiration relative to the unmodified crop. Grain carbon accumulation was initiated after anthesis during Day 200 of the year.

To assess potential impacts on growth and yield of reducing maintenance respiration by slowing protein turnover, we extended a simple whole-plant carbon balance model (Amthor and McCree, 1990). The model (Supplemental Appendix S2) simulates daily photosynthesis (CO_2_ assimilation) of a generic cereal crop from the seasonal pattern of mid-latitude solar irradiance, leaf area growth, and interception of photosynthetically active radiation. A fraction of each day’s photosynthate is consumed in maintenance respiration with the remainder used for growth of new biomass, including growth respiration. Biomass carbon accumulation is partitioned among four plant compartments, including grains, with mobilization and translocation of some preanthesis vegetative biomass to postanthesis grain growth. The crop was simulated from emergence to physiological maturity, comparing two maintenance respiration scenarios: a baseline unmodified crop and one with a 6.5% slower specific maintenance respiration rate achieved by a 90% reduction in turnover rate of 15 high-cost enzymes from an Arabidopsis dataset (Li et al., 2017; Supplemental Table S3). (We took this as an example of what engineering might enable.) All other model parameters were unchanged. The reduced-protein-turnover crop accumulated 2.4% more total biomass carbon and 2.9% more grain carbon (Figure 5). The model did not account for greater grain number (sink strength) that could result from the slightly larger simulated pre-anthesis biomass accumulation (Fischer, 2008) due to lower maintenance respiration, which could favor more grain growth. The 2.4% biomass carbon increase matches our analysis of generic relationships between maintenance respiration, growth respiration, and photosynthesis (Amthor et al., 2019) and is broadly consistent with the biomass increase modeled previously, with somewhat different assumptions, for reduced turnover of two high-cost thiamin synthesis enzymes (Hanson et al., 2018). As the yearly rate of increase in genetic yield potential in high-yield cropping systems such as hybrid maize has now slowed to <1%, perhaps to as little as 0.17% (Di Matteo et al., 2016; Rizzo et al., 2022), the potential genetic gains from engineering slower protein turnover could contribute usefully to maintaining year-on-year yield advances.

## Conclusion

Having up to now treated expansion and destruction of respiratory energy demand separately, we end by considering them together. We showed that destroying 6.5% of the maintenance energy demand by slowing enzyme turnover could increase biomass accumulation by 2.4% (Figure 5). For a maize crop with a total biomass of 24 tonnes ha^−1^, this is 0.58 tonnes ha^−1^, equivalent to 0.81 tonnes ha^−1^ hexose. This extra hexose would in principle be available to support new energy demands. Above, we estimated that 0.53 tonnes ha^−1^ hexose is needed to add 10% suberin or sporopollenin to a maize crop’s roots; this hexose cost would be more than covered by destroying 6.5% of maintenance demand. The yield penalty and economic knock-on effect (Figure 3D) of engineering suberin or sporopollenin accumulation might thus be avoided by stacking this trait with slower enzyme turnover. Similarly, in the lowest-cost diazotroph BNF scenario, where the cost of fully replacing nitrate N is 1.2 tonnes ha^−1^ hexose (Figure 4C), two-thirds of this cost could be covered by a 6.5% maintenance demand destruction. Thus, while respiratory energy demand destruction can stand alone as a crop improvement strategy, it could also be powerfully combined with strategies that address environmental objectives, thereby mitigating the yield sacrifices that these strategies imply.

ADVANCES

- Synthetic biology advances now make it possible, in principle, to increase soil carbon sequestration, enhance BNF, and increase crop yields by cutting respiratory carbon loss.
- The potential scalability and benefits of these and similar interventions with respect to energy use, the environment, and the economy can be modeled to prioritize research projects.
- Biomass synthesis costs can be calculated using the convenient KBase app.

OUTSTANDING QUESTIONS

- To what extent, if any, does the carbon supply from photosynthesis (source) increase to match the added demand (sink) from bolt-on pathways such as recalcitrant polymer biosynthesis and N fixation?
- How much C can realistically be sequestered in soil by engineering recalcitrant polymers in roots, how long do such polymers persist in soil, and how do they affect soil properties?
- How much can N fixation by plant-associated diazotrophs realistically contribute to crop N budgets, and is this contribution associated with lower yield potential?
- How far can the in vivo lifespan of enzymes that are prone to catalytic self-inactivation be extended by continuous directed evolution in a microbial platform?
- How much energy and biomass can realistically be saved by respiratory demand destruction, and can such savings be made without detriment to crop performance?

## Supplemental data

The following materials are available in the online version of this article.

**Supplemental Table S1.** Experimental estimates for crop leaves or roots of the contribution of protein turnover to maintenance respiration or to dark respiration, depending on the study.

**Supplemental Table S2.** Estimated hexose costs of sporopollenin, suberin, and other types of biomass.

**Supplemental Table S3.** Turnover cost calculation for an unmodified crop and an engineered crop with 90% reduced turnover rate of 15 enzymes.

**Supplemental Appendix S1.** Nitrogen acquisition and reduction costs.

**Supplemental Appendix S2.** Crop modeling of reduced protein turnover.

## Supplementary Material

kiac493_Supplementary_Data

## Notes

All authors conceived and designed the article and contributed to writing the article and to the calculations that it contains. A.D.H. agrees to serve as author responsible for contact and ensures communication.

The author responsible for distribution of materials integral to the findings presented in this article in accordance with the policy described in the Instructions for Authors (https://academic.oup.com/plphys/pages/general-instructions) is: Andrew D. Hanson (adha@ufl.edu).
