# Supplementary material for: Respiratory energy demands and scope for demand expansion and destruction
Source: Plant Physiol. 2022 Oct 22;191(4):2093–103. doi: 10.1093/plphys/kiac493 (PMC10069906; doi:10.1093/plphys/kiac493)
Supplement: kiac493_Supplementary_Data [file kiac493_supplementary_data.pdf]

Supplemental Appendix S1

Nitrate reduction and ammonium assimilation costs

NO_3^- reduction cost per g-atom N (Noctor and Foyer, 1998):

	moles hexose	g hexose
1 NADH	0.09	16
3 NADPH	0.26	47

NO_3^- uptake cost per g-atom N (Miller and Smith, 1996):

	moles hexose	g hexose
2 ATP	0.07	13

NH_4^+ assimilation into glutamate per g-atom N (Noctor and Foyer, 1998):

	moles hexose	g hexose
1 ATP	0.04	6
1 NADPH	0.09	16

<u>Total costs</u>

NO_3^- uptake and assimilation: 98 g hexose g-atom^{-1} N = 7.0 g hexose g^{-1} N

NH_4^+ assimilation: 22 g hexose g-atom^{-1} N = 1.6 g hexose g^{-1} N

[NH_4^+ uptake assumed to have no ATP cost (Forde and Clarkson, 1999)]

N_2 to NH_4^+ direct cost (excluding cost of nitrogenase or of maintaining diazotrophic microbes)

Reduction cost per g-atom N (Seefeldt et al., 2018):

	moles hexose	g hexose
8 ATP*	0.29	51
2 NADPH (4 e^-)	0.17	31

*In high-O_2 environments, up to 20 ATP (Batista and Dixon 2019) → 0.71 moles or 129 g hexose

Transport costs (from microbe to plant) per g-atom N (Pate and Layzell, 1990):

	moles hexose	g hexose
2 ATP	0.07	13

<u>Total costs</u>

N_2 reduction *in planta*, assimilation: 104 g hexose g-atom^{-1} N = 7.4 g hexose g^{-1} N

In high-O_2: 182 hexose g-atom^{-1} N = 13 g hexose g^{-1} N

N_2 reduction by diazotrophs, transfer to plant, assimilation: 117 g hexose g-atom^{-1} N = 8.4 g hexose g^{-1} N

In high-O_2: 195 hexose g-atom^{-1} N = 14 g hexose g^{-1} N

Yield penalty estimates (based on above-ground biomass)

Maize grain yield = 11.2 tonnes ha^{-1} (178 bushels acre^{-1}) (Schnitkey et al., 2022)

Harvest index = 0.5 → 11.2 tonnes ha^{-1} stover (Hütsch and Schubert, 2017)

Grain protein content = 10% (Flint-Garcia et al., 2009) → (× 1/6.25) 1.6% N → 180 kg N ha^{-1}

Stover protein content = 4% (Li et al., 2014; NHI ≈70% Mueller et al., 2019) → 70 kg N ha^{-1}

Therefore total crop N content = 250 kg N ha^{-1}

N application rate = 200 kg N ha^{-1} (Davies et al., 2020, Ciampitti and Vyn, 2014)

Applied N recovery in crop = 60% (Ciampitti and Vyn, 2014) = 120 kg ha^{-1}

[Crop N from other sources = (250-120) 130 kg ha^{-1}]

Therefore N from fertilizer to be replaced by BNF = 120 kg ha^{-1}

<u>For soil diazotroph microbes</u>

Assume 50% of fixed N is transferred to crop → 240 kg ha^{-1} required

1. Assume 100% of photosynthate exuded from roots is transferred to diazotrophs, and 100% of this is used to support nitrogenase function

Hexose costs

- 120 kg N ha^{-1} taken up as NO_3^- @ 7.0 g hexose g^{-1} N = 840 kg hexose ha^{-1}
- 240 kg N ha^{-1} fixed by soil microbes @ 8.4 g hexose g^{-1} N = 2016 kg hexose ha^{-1}
  - @ 14 g hexose g^{-1} N = 3360 kg hexose ha^{-1}

Differences: 1176 or 2520 kg hexose ha^{-1} (sugar exudate required = 5 or 11% of aerial biomass)

@ 1.41 g hexose per g maize grain, yield penalties are:

- 0.8 or 1.8 tonnes ha^{-1}
- @ grain yield 11.2 tonnes ha^{-1} = 7% or 16%

2. Assume 50% of photosynthate exuded from roots is transferred to diazotrophs, and 50% of this is used to support nitrogenase function

Hexose costs

- 120 kg N ha^{-1} taken up as NO_3^- @ 7.0 g hexose g^{-1} N = 840 kg hexose ha^{-1}
- 240 kg N ha^{-1} fixed by soil microbes @ (8.4 × 4) g hexose g^{-1} N = 8064 kg hexose ha^{-1}
  - @ 14 g hexose g^{-1} N = 13440 kg hexose ha^{-1}

Differences: 7224 or 12600 kg hexose ha^{-1} (sugar exudation required = 32 or 56% of aerial biomass)

@ 1.41 g hexose per g maize grain, yield penalties are:

- 5.1 or 8.9 tonnes ha^{-1}
- @ grain yield 11.2 tonnes ha^{-1} = 46% or 79%

Average value for total carbon loss from roots to rhizosphere is up to ~20%, of which only a fraction is exudate (Hütsch et al., 2002; Nguyen, 2003).

<u>Plant-encoded leaf nitrogenase</u>

All fixed N available to crop → 120 kg ha^{-1} required

Hexose cost of plant nitrogenase assimilation of 120 kg N:

- 120 kg N ha^{-1} @ 7.4 g hexose g^{-1} N = 888 kg hexose ha^{-1}
- 120 kg N ha^{-1} @ 13 g hexose g^{-1} N = 1560 kg hexose ha^{-1}

Difference from NO_3^-: 48 kg hexose ha^{-1} or 720 kg hexose ha^{-1}

@ 1.41 g hexose per g maize grain, yield penalty is:

- 0.03 tonnes ha^{-1} or 0.51 tonnes ha^{-1}
- @ grain yield 11.2 tonnes ha^{-1} = 0.3% or 5%

Appendix S1 References

Bueno-Batista M, Dixon R (2019) Manipulating nitrogen regulation in diazotrophic bacteria for agronomic benefit. Biochem Soc Trans **47:** 603–614

Ciampitti IA, Vyn TJ (2014) Understanding global and historical nutrient use efficiencies for closing maize yield gaps. Agron J **106:** 2107–2117

Davies B, Coulter JA, Pagliari PH (2020) Timing and rate of nitrogen fertilization influence maize yield and nitrogen use efficiency. PLoS One **15:** e0233674

Forde BG, Clarkson DT (1999) Nitrate and ammonium nutrition of plants: Physiological and molecular perspectives. Adv Bot Res **30:** 1–90

Hütsch BW, Augustin J, Merbach W (2002) Plant rhizodeposition an important source for carbon turnover in soils. J Plant Nutr Soil Sci **165:** 397–407

Hütsch BW, Schubert S (2017) Harvest index of maize (*Zea mays* L.): are there possibilities for improvement? Adv Agron **146:** 37–82

Li H Y, Xu L, Liu WJ, Fang MQ, Wang N (2014) Assessment of the nutritive value of whole corn stover and its morphological fractions. Asian Australas J Anim Sci **27:** 194–200

Miller AJ, Smith SJ (1996) Nitrate transport and compartmentation in cereal root cells. J Exp Bot **47:** 843–854

Mueller SM, Messina CD, Vyn TJ (2019) Simultaneous gains in grain yield and nitrogen efficiency over 70 years of maize genetic improvement. Sci Rep **9:** 9095

Nguyen C (2003) Rhizodeposition of organic C by plants: mechanisms and controls. Agronomie **23:** 375–396

Noctor G, Foyer CH (1998) A re-evaluation of the ATP:NADPH budget during C3 photosynthesis: a contribution from nitrate assimilation and its associated respiratory activity? J Exp Bot **49:** 1895–1908

Pate JS, Layzell DB (1990) Energetics and biological costs of nitrogen assimilation. *In* BJ Miflin, PJ Lea, eds, *The Biochemistry of Plants*, Vol. 16, Intermediary Nitrogen Metabolism. Academic Publishing, San Diego, CA, pp 1–42

Schnitkey G, Zulauf C, Swanson K, Paulson N, Coppess J, Baltz J (2022) Perspectives on national U.S. corn yields for productivity and down-side yield risk." farmdoc daily (12):103, Department of Agricultural and Consumer Economics, University of Illinois at Urbana-Champaign

Seefeldt LC, Hoffman BM, Peters JW, Raugei S, Beratan DN, Antony E, Dean DR (2018) Energy transduction in nitrogenase. Acc Chem Res **51:** 2179–2186

Supplemental Appendix S2

Formulas Used to Calculate Reduced Costs of Protein Turnover

FW, fresh weight; DW, dry weight

$$Enzyme\ abundance\ (nmol\ g^{-1}FW) = \frac{(Protein\ abundance\ (\%) * 0.01) * 15\ mg\ g^{-1}FW * 10^{6}}{Molecular\ mass\ (g\ mol^{-1})}$$

$$Turnover\ rate\ (nmol\ g^{-1}\ FW\ d^{-1}) = Enzyme\ abundance\ (nmol\ g^{-1}\ FW) * K_D\ (d^{-1})$$

This was multiplied × 0.1 for a 90% turnover rate reduction

$$Protein\ breakdown\ and\ synthesis\ (ATP\ molecule^{-1}) = 6.3\ ATP\ residue^{-1} * No.of\ residues$$

$$ATP\ cost\ of\ turnover\ (\mu mol\ g^{-1}FW\ d^{-1}) = Turnover\ rate\ (nmol\ g^{-1}\ FW\ d^{-1}) * Protein\ breakdown\ and\ synthesis\ (ATP\ molecule^{-1}) * 10^{-3}$$

$$Hexose\ cost\ of\ turnover\ (\mu mol\ g^{-1}\ FW\ d^{-1}) = \frac{ATP\ cost\ of\ turnover\ (\mu mol\ g^{-1}FW\ d^{-1})}{28}$$

$$Hexose\ cost\ of\ turnover\ (\mu mol\ g^{-1}\ DW\ d^{-1}) = \frac{Hexose\ cost\ of\ turnover\ (\mu mol\ g^{-1}\ FW\ d^{-1})}{0.12}$$

$$Hexose\ cost\ of\ turnover\ (mg\ g^{-1}\ DW\ d^{-1}) = Hexose\ cost\ of\ turnover\ (\mu mol\ g^{-1}\ DW\ d^{-1}) * 180\ g\ mol^{-1} * 10^{-3}$$

Crop Model Description and Parameterization

The model is a simplified representation of crop carbon balance meant to reflect general quantitative relationships between photosynthesis, respiration, and plant carbon accumulation. In particular, the model partitions daily photosynthate carbon between respiration to support maintenance metabolism and a 'remainder' used for growth of new biomass, with the new growth partitioned between plant parts, culminating in growth of seeds only after anthesis. This method of modeling respiration can be considered a growth-and-maintenance respiration approach (Amthor, 2000; Cannell and Thornley, 2000). The model is applied to uniform, unstressed crops. All standing crop mass and flux values are for carbon rather than fresh or dry total phytomass.

This study's focus was the maintenance respiration coefficient *m* (g C [respired] g^{-1} C [plant] d^{-1}, or simply d^{-1}). The value of *m* in a crop modified for slower protein turnover was reduced by 6.5% relative to an unmodified crop. The change in protein half-life was assumed to have no effect on plant composition, i.e., the protein content was unchanged and therefore the growth yield Y_G (carbon appearing in new biomass per unit of photosynthate carbon used in growth processes) was unchanged. If protein turnover were slowed in actual plants, there might be a small reduction in crop nitrogen concentration and corresponding increase in Y_G due to reduced biosynthesis of enzymatic machinery needed for protein breakdown and re-synthesis.

Model Structure

The model was parameterized for a crop season from emergence during day of year (DoY) 121 to physiological maturity during DoY 250 at latitude 42°N. Other seasons and locations result in different specific outcomes, but the principles of the model are expected to apply generally. The model time step is 1 day (24 h). A crop developmental state D is defined as the fraction of time elapsed from emergence to maturity, covering the interval [0,1]. In this case it is defined by

$$D = (J - 121) / (250 - 121)$$

where J is day of the year. Values of several modeled physiological variables are functions of D.

Solar Radiation and Photosynthesis (CO_2 assimilation)

Daily extraterrestrial solar irradiance ($H_{h,o}$, MJ m^{-2} d^{-1}) was calculated based on latitude. An average all-sky global atmospheric transmittance of solar radiation (τ_{atm}) of 54% (Trenberth et al., 2009) was used each day over the season; the only day-to-day variation in radiation was thus due to Earth-Sun geometry. An average PAR fraction of downwelling solar radiation at the crop surface (F_{PAR}) of 48% (Amthor, 2010) was used to calculate daily PAR incident on the crop (Appendix Figure 1).

Interception of PAR by the canopy (F_{PAR}, fraction [0,1]) is given by:

$$F_{PAR} = (1 - e^{-k\,L})$$

where the extinction coefficient k is 0.6 in the model and L is crop leaf area index (LAI, m^2 m^{-2}). LAI is the product of whole-crop leaf mass (W_{leaf}, g C m^{-2} ground) and specific leaf area s (= 0.040 m^2 (g leaf C)$^{-1}$), which is held constant in the model.

Photosynthesis (P, g C m^{-2} d^{-1}) is the product $\varepsilon\ P_{capacity}\ F_{PAR}\ f_{PAR}\ \tau_{atm}\ H_{h,o}$, where ε is photosynthetic carbon assimilation (photosynthesis less photorespiration) per unit PAR intercepted (= 1.5 g C (MJ intercepted PAR)$^{-1}$ in the model; Loomis & Amthor, 1999). To account for loss of canopy photosynthetic capacity due to leaf senescence toward the end of the season, a relative photosynthetic capacity factor ($P_{capacity}$, [0,1]) is included (Appendix Figure 2).

Abscission of senesced leaves is not simulated, leading to slightly larger leaf biomass carbon at the end of the season than would be measured in the field.

Maintenance Respiration (CO_2 release)

Maintenance respiration rate (R_M, g C m^{-2} d^{-1}) is the product mW, where m is the maintenance coefficient, or specific maintenance respiration rate (g C (g C in biomass)$^{-1}$ d^{-1}; more simply d^{-1}), and W is plant carbon content (g C m^{-2} ground). There are four plant compartments in the model (see below) each with a separate m and W. Experimental data indicates m declines during the season (e.g., McCree and Silsbury, 1978; McCree, 1982, 1983; Stahl and McCree, 1988). To accommodate this, an initial date-of-emergence value of m (m_0) is defined for each plant compartment with values for each subsequent day given by:

$$m = m^*\ m_0$$

where m^* is a time-dependent ontogenetic coefficient (Appendix Figure 2). As implemented for the unmodified crop, i.e., not engineered to reduce protein turnover, m_0 values were 0.029, 0.022, 0.015, and 0.006 d^{-1} for leaf blades, roots, 'other,' and seeds, where 'other' is a mixture of leaf sheath, stem, and spike tissues. Maintenance respiration in seeds is always less than the value associated with m_0 = 0.006 d^{-1} because m^* is significantly less than unity by the time of anthesis, which in the model was at day of year 199 when D reached 0.60, the trigger for anthesis. For the reduced-protein-turnover engineered crop, m_0 was reduced by 6.5% in all organs.

Whole-plant R_M is the sum of mW for all four plant compartments during each day.

The modeled decline in m with crop development might be considered an artificial 'fix' to a weakness of the growth-and-maintenance respiration modeling approach (see Thornley, 2011). Alternatively—and the view taken herein—the decline in m with development reflects a decline in the fraction of biomass undergoing turnover. In leaves, this is reflected in declining nitrogen concentration with age in expanded leaves. In the case of seeds, this is related to the quantitatively significant production and accumulation of long-term storage compounds—be they carbohydrates, proteins and/or lipids—rather than metabolically active cellular fractions.

Growth (Biomass Carbon Accumulation) and Growth Respiration (CO_2 Release)

Each daily value of the difference $P - R_M$ is used in growth processes (in this model setup, R_M was always less than P*)*. The associated growth respiration rate (R_G, g C m^{-2} d^{-1}) is given by:

$R_G = (1 - Y_G)(P - R_M)$,

where Y_G is the yield (or carbon-use efficiency) of the growth processes. It is expressed as g C in new biomass per g C in substrate used for growth, with that substrate including the carbon incorporated into new biomass. Y_G is set to a constant value of 0.72 (McCree and Silsbury, 1978; McCree, 1982; Stahl and McCree, 1988; Cannel and Thornley, 2000; Lötscher et al., 2004) as a simplification, though it can (does) change during the lifespan of actual crops (McCree, 1988; Amthor, 2010) because the composition of biomass being synthesized changes. For example, a change from relatively low-protein, low-lipid vegetative growth early in the season to growth of high-protein and/or high-lipid seeds later in the season can cause Y_G to decline, whereas a transition to formation of high-carbohydrate seeds or tubers later in the seasonal can cause Y_G to increase. The whole-plant Y_G used in this model includes all processes underlying growth such as biosynthesis in growing cells/tissues, source-sink transport of carbon substrate (short-term reserve mobilization and phloem loading), and nitrogen uptake and assimilation. In models simulating direct, local biosynthesis values of Y_G usually will be (considerably) larger (Penning de Vries et al., 1974; Amthor, 2000; Cannell and Thornley, 2000).

Following from the above, daily growth (ΔW, g C in new biomass m^{-2} d^{-1}) is given by:

$\Delta W = Y_G (P - R_M)$.

The carbon in new biomass is a combination of compounds with various longevities ranging from 'permanent' cell wall components to short-term storage compounds.

Nonstructural carbohydrate amount is not tracked in the model. Rather, it is assumed that the crop is in a relatively steady state of carbon acquisition in photosynthesis and use in growth and respiration. As such, the model is applicable to non-stressful environments with gradual and small day-to-day environmental changes. Further, root death, exudation of carbon from roots, leaf abscission, and herbivory were ignored in the model, as were volatilization and leaching of carbon from the plant. These can be significant amounts in some circumstances.

Partitioning ΔW Among Plant Parts

Whole-crop carbon content at emergence (W_0, g C m^{-2} ground) was set to 0.1 g C m^{-2} ground and divided between W_{leaf} (80%) and the root compartment (W_{root}, g C m^{-2} ground) (20%). Partitioning of ΔW among plant fractions during each simulated day after emergence then followed simple partitioning coefficient rules based on D (Appendix Figure 3). In this model application, the magnitude and temporal dynamics of the partitioning coefficients were meant to be typical of cereal crops.

Grain filling and sink strength

Modeled grain carbon (W_{grain}, g C m^{-2} ground) came from two sources, as it does in actual crops: current (post-anthesis) photosynthesis and mobilization of pre-anthesis protein and carbohydrates in vegetative plant parts. The current photosynthesis component is added daily in the amount p_{grain} ΔW (for $p_{grain} > 0$), where p_{grain} is the grain photosynthate carbon partitioning coefficient, which is zero prior to anthesis and increases rapidly to 1.0 shortly after anthesis and does not respond to bioengineered change in the maintenance respiration coefficient in the model. The mobilization component simulates a conservative 15% contribution to total grain carbon (Gebbing et al., 1999) so that each day during grain filling an amount of carbon equal to $0.15\, p_{grain} \Delta W / (1 - 0.15)$ is transferred from the W_{leaf} and W_{other} (g C m^{-2} ground, a mixture of leaf sheath, stem, and spike tissues) plant parts, equally divided between the two.

To account for the respiratory cost of mobilization and then biosynthesis of new grain tissue from the mobilized carbon, an amount of carbon equal to 50% of the carbon transferred from W_{leaf} and W_{other} to W_{grain} is added to daily growth respiration. That respiratory carbon is removed from W_{leaf} and W_{other}. This represents the measured 67% efficiency of mobilized carbon retention in new grain biomass during grain filling in wheat (Gebbing et al., 1999). Neither the 15% of grain carbon accumulation arising from mobilization of pre-anthesis carbon accumulation nor the 67% efficiency of converting the mobilized carbon into seed biomass carbon were affected by the reduced maintenance respiration coefficient in the modeled bioengineered crop.

Although the model simulated increased pre-anthesis biomass carbon accumulation in the bioengineered crop with smaller maintenance respiration coefficient, it did not simulate greater seed number (and consequent sink strength) that could result from that greater pre-anthesis biomass accumulation (Fischer, 2008).

Key results

The reduced-*m* crop had slightly greater photosynthesis during the season (Appendix Figure 4) due to additional leaf growth and the resulting increase in LAI and PAR interception. Peak LAI for the unmodified crop was 5.40 m^2 m^{-2}, compared to 5.51 m^2 m^{-2} for the reduced-*m* crop. The cumulative increase in carbon assimilation was 9.7 g C m^{-2} , or 0.6% more than the unmodified crop.

Whole-plant maintenance respiration was smaller in the reduced-*m* crop while growth respiration *increased* (Appendix Figures 5 and 6), due to the availability of additional substrate for growth. Total biomass carbon production increased 2.4% and grain carbon production increased 2.9% in response to the 6.5% reduction in *m* (Figure 5).

Appendix S2 References

Amthor JS (2010) From sunlight to phytomass: on the potential efficiency of converting solar radiation to phyto-energy. New Phytol **188:** 939–959

Cannell MGR, Thornley JHM (2000) Modelling the components of plant respiration: some guiding principles. Ann Bot **85:** 45–54

Fischer RA (2008) The importance of grain or kernel number in wheat: a reply to Sinclair and Jamieson. Field Crops Res **105:** 15–21

Gebbing T, Schnyder H, Kühbauch W (1999) The utilization of pre-anthesis reserves in grain filling of wheat. Assessment by steady-state $^{13}CO_2$/$^{12}CO_2$ labelling. Plant Cell Environ **22:** 851–858

Loomis RS, Amthor JS (1999) Yield potential, plant assimilatory capacity, and metabolic efficiencies. Crop Sci **39:** 1584–1596

Lötscher M, Klumpp K, Schnyder H (2004) Growth and maintenance respiration for individual plants in hierarchically structured canopies of *Medicago sativa* and *Helianthus annuus*: the contribution of current and old assimilates. New Phytol **164:** 305–316.

McCree KJ (1982). Maintenance requirements of white clover at high and low growth rates. Crop Sci **22:** 345-351.

McCree KJ (1983) Carbon balance as a function of plant size in sorghum plants. Crop Sci **23:** 1173–1177

McCree KJ, Silsbury JH (1978). Growth and maintenance requirements of subterranean clover. Crop Sci **18:** 13–18

Stahl RS, McCree KJ (1988) Ontogenetic changes in the respiration coefficients of grain sorghum. Crop Sci **28:** 111–113

Thornley JHM (2011) Plant growth and respiration re-visited: maintenance respiration defined – it is an emergent property of, not a separate process within, the system – and why the respiration: photosynthesis ratio is conservative. Ann Bot **108:** 1365–1380

Trenberth KE, Fasullo JT, Kiehl J (2009) Earth’s global energy budget. Bull Am Meteorol Soc **90**: 311–323

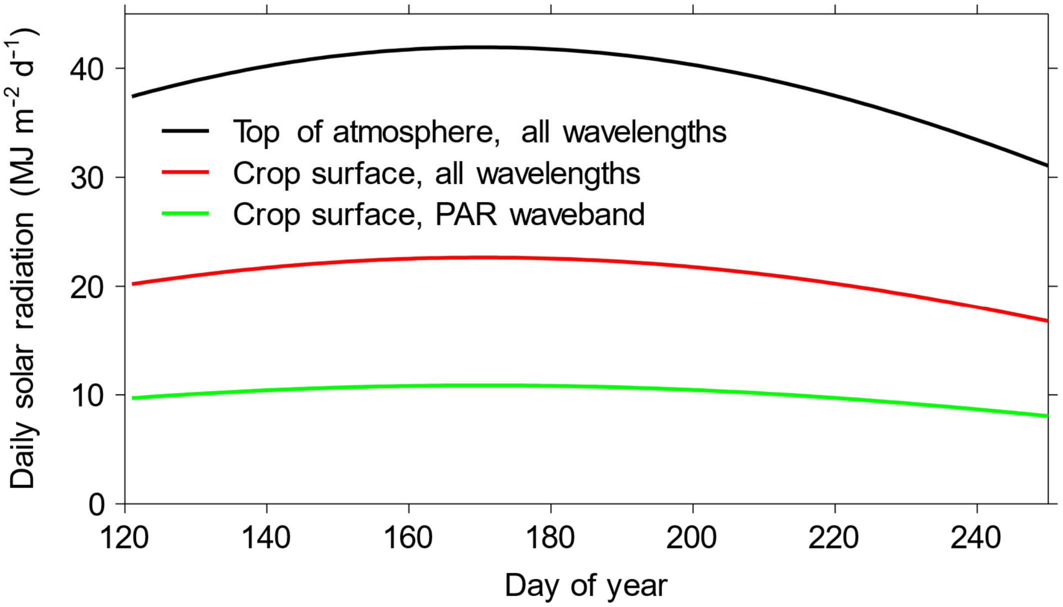

Appendix Figure 1. Modeled daily top-of-atmosphere (extraterrestrial) global solar irradiance ($H_{h,o}$), crop-surface global solar irradiance, and crop-surface global photosynthetically active (PAR, 400–700 nm) radiation during the period day-of-year 120 to 250 at 42°N latitude with all-sky global atmospheric transmittance of solar radiation (τ_{atm}) of 54% and an average PAR fraction of downwelling solar radiation at the crop surface (f_{PAR}) of 48%.

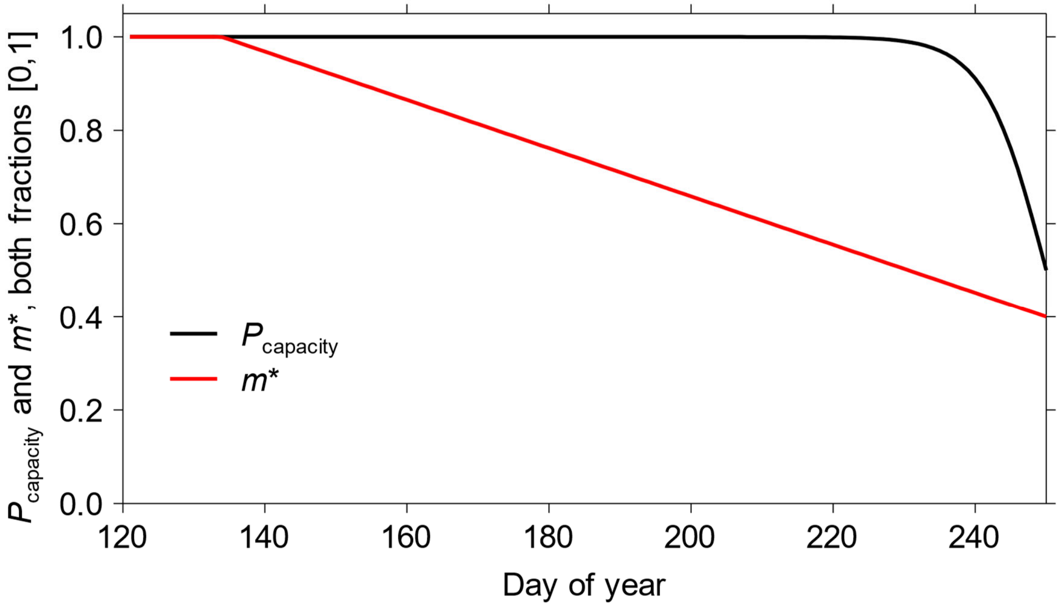

Appendix Figure 2. Seasonal course of relative canopy photosynthetic capacity ($P_{capacity}$) and relative maintenance respiration coefficient (m*), both fractions [0,1].

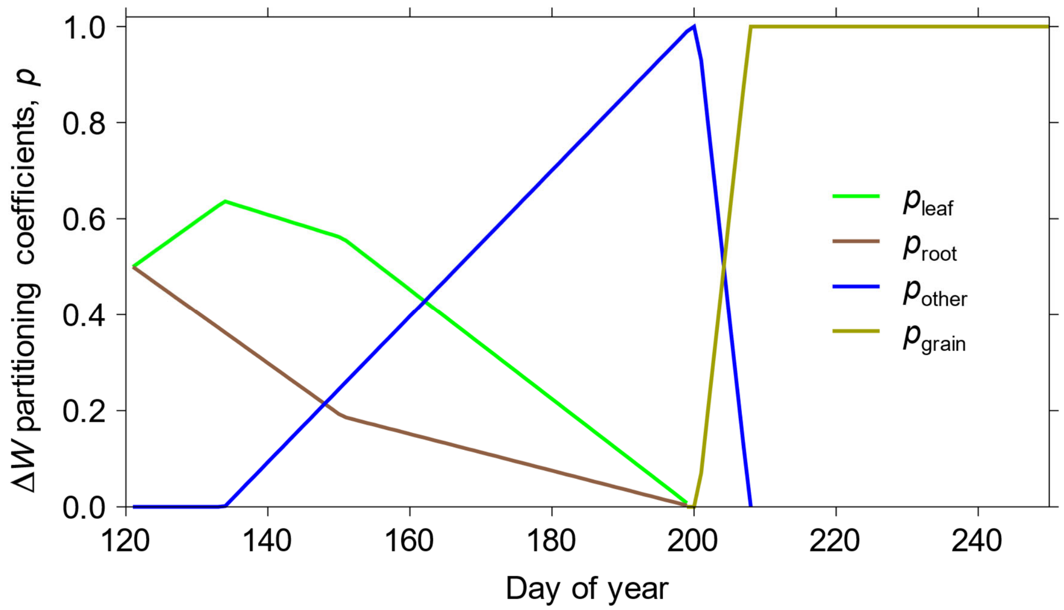

Appendix Figure 3. Partitioning coefficients for new biomass carbon accumulation in leaf blades (p_{leaf}), roots (p_{root}), seeds (p_{grain}), and 'other' plant parts (p_{other}). The 'other' fraction includes stems, leaf sheaths, and spikes (the non-seed fraction of the reproductive organs). Anthesis was reached on day 205 in the model after which grain growth quickly became the dominant carbon sink.

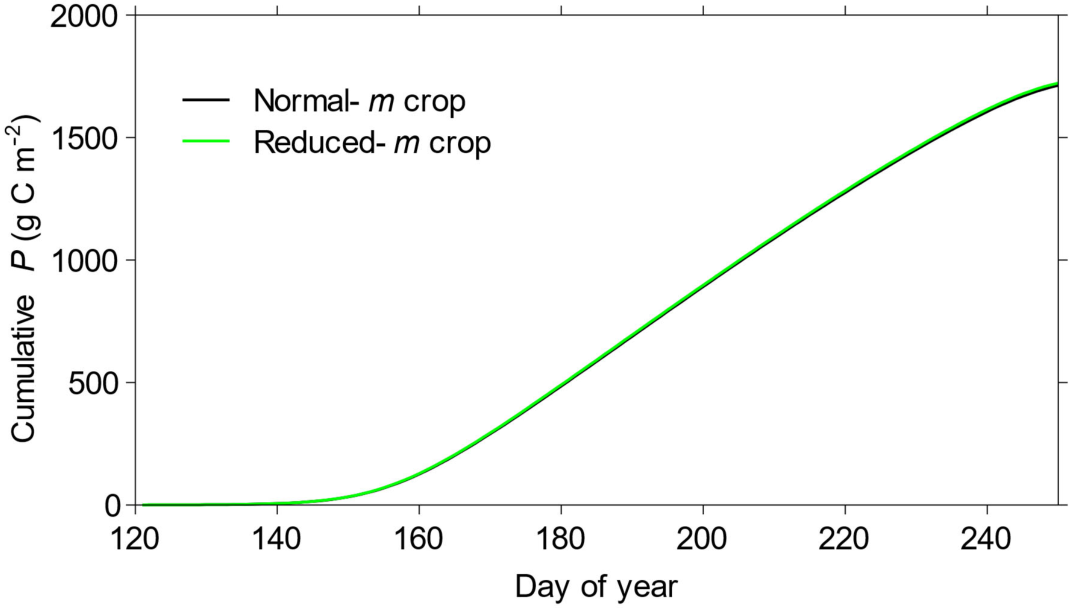

Appendix Figure 4. Simulated whole-crop cumulative photosynthesis for the normal (unmodified) crop and the reduced-*m* crop with a 6.5% smaller maintenance respiration coefficient. Area is ground area covered by the crop.

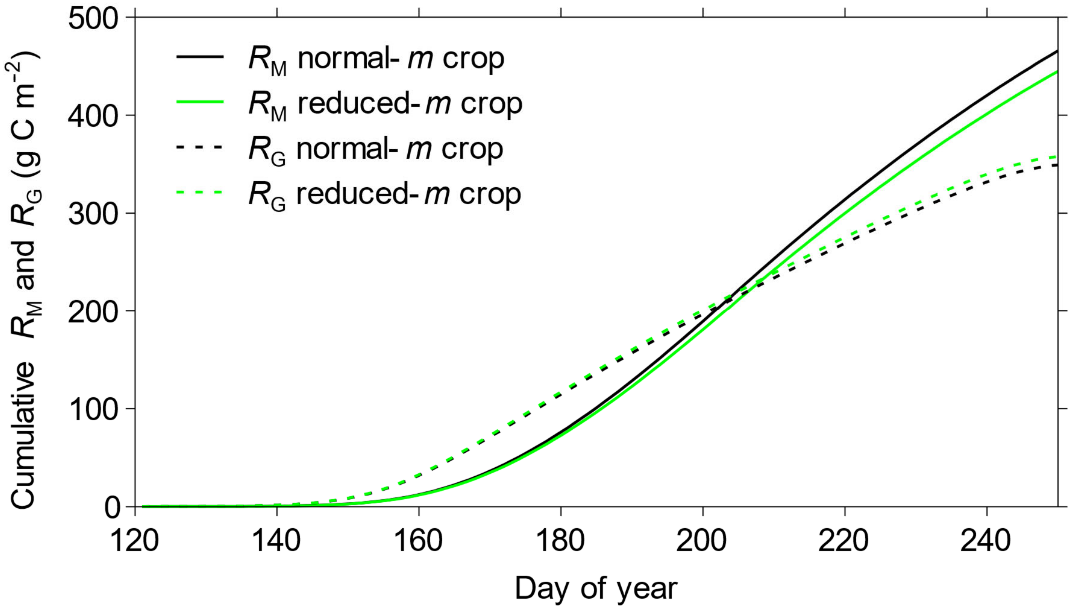

Appendix Figure 5. Simulated cumulative maintenance respiration (R_M, solid lines) and growth respiration (R_G, dashed lines) for the unmodified crop (black lines) and the crop with 6.5% reduction in maintenance respiration coefficient (green lines). Area is ground area covered by the crop. Note that growth respiration increased in the reduced-*m* crop.

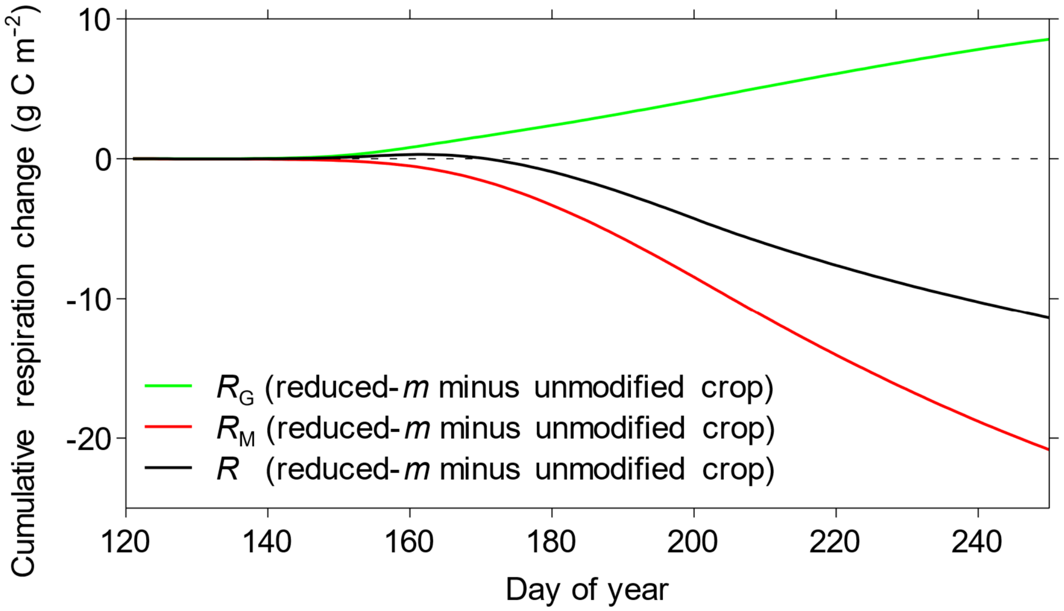

Appendix Figure 6. Cumulative difference in total respiration (R), maintenance respiration (R_M), and growth respiration (R_G) between the reduced-*m* crop and the unmodified crop. Value plotted is reduced-*m* crop minus the unmodified crop. A positive value indicates greater respiration in the reduced-*m* crop. Area is ground area covered by the crop.

Supplemental Table S1. Experimental estimates for crop leaves or roots of the contribution of protein turnover to maintenance respiration or to dark respiration, depending on the study. These estimates involved a wide range of assumptions and methodologies. As mature leaf respiration supports phloem loading as well as maintenance, the contribution of protein turnover expressed on this basis is necessarily an underestimate of its contribution to maintenance respiration.

Plant/organ	% of Respiration	Reference
Barley, fully expanded flag leaves	10	de Visser et al. (1992)
Bean, fully expanded leaves	66	de Visser et al. (1992)
	17-21	Bouma et al. (1994)
	41-61	Noguchi et al. (2001)
Bean, expanding leaves	17-37	Bouma et al. (1994)
Potato, expanded leaves (field)	64	de Visser et al. (1992)
Potato, expanded leaves (greenhouse)	76	de Visser et al. (1992)
Ryegrass, mature leaves	27-36	Barneix et al. (1988)
	68	de Visser et al. (1992)
Wheat, fully grown leaves	34	Zagdańska (1995)
Pasture grasses, roots	22-30	Scheurwater et al. (2000)
**Mean ± SD**	**43 ± 22** .	

References

Barneix AJ, Cooper HD, Stulen I, Lambers H (1988), Metabolism and translocation of nitrogen in two *Lolium perenne* populations with contrasting rates of mature leaf respiration and yield. Physiol Plant **72:** 631–636

Bouma TJ, De Visser R, Janssen J, De Kock M, Van Leeuwen P, Lambers H (1994), Respiratory energy requirements and rate of protein turnover in vivo determined by the use of an inhibitor of protein synthesis and a probe to assess its effect. Physiol Plant **92:** 585–594

De Visser R, Spitters CJT, Bouma TJ (1992) Energy costs of protein turnover: theoretical calculation and experimental estimation from regression of respiration on protein concentration of full-grown leaves. *In* Lambers H, van der Plas LHW, eds, *Molecular, Biochemical and Physiological Aspects of Plant Respiration*, SPB Acad Publishing, The Hague, pp 493–508

Noguchi K, Go C-S, Miyazawa S-I, Terashima I, Ueda S, Yoshinari T (2001) Costs of protein turnover and carbohydrate export in leaves of sun and shade species. Funct Plant Biol **28:** 37–47

Scheurwater I, Dünnebacke M, Eising R, Lambers H (2000) Respiratory costs and rate of protein turnover in the roots of a fast-growing (*Dactylis glomerata* L.) and a slow-growing *(Festuca ovina* L.) grass species. J Exp Bot **51:** 1089–1097

Zagdańska B (1995), Respiratory energy demand for protein turnover and ion transport in wheat leaves upon water deficit. Physiol Plant **95:** 428–436

Supplemental Table S2. Estimated hexose costs of sporopollenin, suberin, and other types of biomass.

	Sporopollenin ($C_{200}H_{312}O_{64}$)*				Suberin ($C_{125}H_{224}O_{29}$)*			
	ATP	NAD[P]Hs	Hexoses	CO_2 lost	ATP	NAD[P]Hs	Hexoses	CO_2 lost
Per polymer molecule	-36	24	-60	-88	-56	-78	-31	-68
Number of polymer molecules in 1 g polymer	1.79E+20	1.79E+20	1.79E+20	1.79E+20	2.75E+20	2.75E+20	2.75E+20	2.75E+20
Energy cost per 1 g polymer	-6.444E+21	4.296E+21	1.074E+22	-1.575E+22	-1.54E+22	-2.14E+22	8.51E+21	-1.8674E+22
Extra hexoses to balance energy budget			1.76333E+19				2.47E+21	
Total transport and polymerization cost per 1 g polymer			1.84867E+20				2.51E+20	
Total hexose cost per 1 g polymer			1.09E+22				1.12E+22	
Total g hexose per 1 g polymer			3.27E+00				3.36E+00	

Biomass type	g hexose per 1 g biomass
C4 stover biomass	1.19
Protein	1.83
Triacylglycerol (triolein)	3.05
Sporopollenin	3.27
Suberin	3.36

*The empirical formulae of the average sporopollenin (C200H312O64) and suberin (C125H224O29) molecules (Figure 3, A and B) were used to calculate the number of molecules to make up 1 g of polymer (Row 5) and the number of ATP, NAD[P]H, and hexose molecules required to synthesize the polymer components (Row 4). Additional hexoses required to balance the ATP or NAD[P]H requirements from the biosynthesis were calculated and factored in (Row 7). The additional transport and polymerization costs of suberin (29 ATP per molecule), and sporopollenin (40 ATP per molecule with spare NAD[P]H from backbone reduction) (Row 8). The total hexose cost for biosynthesis, transport and polymerization was calculated (Row 9) and converted to g hexose per 1 g of polymer (Row 10). A P/O ratio of 1.385 ATP/NAD[P]H was used for leftover NAD[P]H.

Supplemental Table S3. Turnover cost calculation for an unmodified crop and an engineered crop with 90% reduced turnover rate of 15 enzymes.

AGI	Description	EC	Total Flux (µmol g^{-1} FW d^{-1})	Flux proportion	Flux (µmol g^{-1} FW d^{-1})	Molecular mass (Da)	as % total leaf protein	Enzyme abundance (nmol g^{-1} FW)	In vivo K_{cat} (d^{-1})	K_D (d^{-1})	CCR
15 Arabidopsis enzymes with highest ATP costs (Li et al., 2017)											
ATCG00490	RBCL Ribulose-bisphosphate carboxylase	4.1.1.39	600	1	600	-	-	26.059	23025	0.052	441317
AT2G39730	RCA Rubisco activase	4.1.1.39	n.d.	n.d.	n.d.	51981	2.4281	7.007	n.d.	0.161	n.d.
ATCG00480	ATPB ATP synthase beta subunit	7.1.2.2	n.d.	n.d.	n.d.	53934	1.6539	4.600	n.d.	0.112	n.d.
ATCG00120	ATPA ATPase alpha subunit	7.1.2.2	n.d.	n.d.	n.d.	55328	1.562	4.235	n.d.	0.079	n.d.
AT5G54770	THI4 Thiazole synthase	2.4.2.60	0.00104274	1	0.00104274	-	-	0.540	2	1.931	1
AT1G20340	DRT112 DNA-damage resistance protein		n.d.	n.d.	n.d.	16984	2.3008	20.320	n.d.	0.082	n.d.
AT3G45140	LOX2 lipoxygenase	1.13.11.12	n.d.	n.d.	n.d.	102046	0.2303	0.339	n.d.	0.180	n.d.
AT4G20360	RABE1b EF-Tu translation elongation factor		n.d.	n.d.	n.d.	51630	0.7097	2.062	n.d.	0.079	n.d.
AT5G04140	GLU1 Glutamate synthase 1 (Fd-GOGAT)	1.4.7.1	150	0.89	133.5	-	-	0.233	574140	0.091	6294833
AT5G09660	PMDH2 NAD-dependent malate dehydrogenase	1.1.1.37	n.d.	n.d.	n.d.	37369	0.5478	2.199	n.d.	0.181	n.d.
AT4G38970	FBA2 Fructose-bisphosphate aldolase 2	4.1.2.13	600	0.69	414	-	-	5.257	78750	0.093	849549
ATCG00020	D1 chlorophyll binding protein	1.10.3.9	n.d.	n.d.	n.d.	38937	0.1031	0.397	n.d.	1.081	n.d.
AT4G33010	GLDP1 Gly cleavage complex P-protein 1	1.4.4.2	150	0.63	94.5	-	-	0.239	395455	0.205	1928773
AT5G42650	AOS Cyt p450 CYP74 allene oxide synthase	4.2.1.92	n.d.	n.d.	n.d.	58197	0.1264	0.326	n.d.	0.523	n.d.
AT1G06680	PSBP-1 PSII-P protein		n.d.	n.d.	n.d.	23759	0.9745	6.152	n.d.	0.086	n.d.

	Turnover cost calculation for an unmodified crop (F=1)							Turnover cost calculation with 90% turnover rate reduction (F =0.1)							Comparison turnover costs	
AGI	Turnover rate (nmol g^{-1} FW d^{-1})	No. of residues	Protein breakdown and synthesis (ATP/ molecule)	ATP cost of turnover (µmol g^{-1} FW d^{-1})	Hexose cost of turnover (µmol g^{-1} FW d^{-1})	Hexose cost of turnover (µmol g^{-1} DW d^{-1})	Hexose cost of turnover (mg g^{-1} DW d^{-1})	Turnover rate (nmol g^{-1} FW d^{-1}) reduced by 90%	Number of residues	Protein breakdown and synthesis (ATP/ molecule)	ATP cost of turnover (µmol g^{-1} FW d^{-1})	Hexose cost of turnover (µmol g^{-1} FW d^{-1})	Hexose cost of turnover (µmol g^{-1} DW d^{-1})	Hexose cost of turnover (mg g^{-1} DW d^{-1})	Cost of turnover (% of maintenance) (F=1)	Cost of turnover (% of maintenance) (F=0.1)
ATCG00490	1.360	479	3017.7	4.103	0.1465	1.221	0.2200	0.1360	479	3018	0.4103	0.014652746	0.1221	0.021998656	1.467	0.147
AT2G39730	1.125	474	2986.2	3.359	0.1200	1.000	0.1801	0.1125	474	2986	0.3359	0.011996806	0.1000	0.018011205	1.201	0.120
ATCG00480	0.517	498	3137.4	1.622	0.0579	0.483	0.0869	0.0517	498	3137	0.1622	0.005791152	0.0483	0.008694449	0.580	0.058
ATCG00120	0.335	507	3194.1	1.069	0.0382	0.318	0.0573	0.0335	507	3194	0.1069	0.00381797	0.0318	0.005732046	0.382	0.038
AT5G54770	1.043	349	2198.7	2.293	0.0819	0.682	0.1229	0.1043	349	2199	0.2293	0.008187948	0.0682	0.012292839	0.820	0.082
AT1G20340	1.656	167	1052.1	1.743	0.0622	0.519	0.0934	0.1656	167	1052	0.1743	0.006223432	0.0519	0.009343446	0.623	0.062
AT3G45140	0.061	896	5644.8	0.344	0.0123	0.102	0.0185	0.0061	896	5645	0.0344	0.001229086	0.0102	0.001845267	0.123	0.012
AT4G20360	0.163	476	2998.8	0.488	0.0174	0.145	0.0261	0.0163	476	2999	0.0488	0.001741312	0.0145	0.00261429	0.174	0.017
AT5G04140	0.021	1622	10218.6	0.217	0.0077	0.064	0.0116	0.0021	1622	10219	0.0217	0.000773981	0.0064	0.001162004	0.077	0.008
AT5G09660	0.398	354	2230.2	0.888	0.0317	0.264	0.0476	0.0398	354	2230	0.0888	0.003171875	0.0264	0.004762041	0.317	0.032
AT4G38970	0.487	398	2507.4	1.222	0.0436	0.364	0.0655	0.0487	398	2507	0.1222	0.004363928	0.0364	0.00655171	0.437	0.044
ATCG00020	0.429	353	2223.9	0.955	0.0341	0.284	0.0512	0.0429	353	2224	0.0955	0.003408952	0.0284	0.005117973	0.341	0.034
AT4G33010	0.049	1037	6533.1	0.320	0.0114	0.095	0.0172	0.0049	1037	6533	0.0320	0.001143173	0.0095	0.001716284	0.114	0.011
AT5G42650	0.171	518	3263.4	0.557	0.0199	0.166	0.0298	0.0171	518	3263	0.0557	0.00198759	0.0166	0.002984035	0.199	0.020
AT1G06680	0.532	263	1656.9	0.881	0.0315	0.262	0.0472	0.0532	263	1657	0.0881	0.003146062	0.0262	0.004723287	0.315	0.031
														Sum	7.170	0.717

Reduced specific maintenance respiration rate by 6.453% %
